# Ixonnexin from Tick Saliva Promotes Fibrinolysis by Interacting with Plasminogen and Tissue-Type Plasminogen Activator, and Prevents Arterial Thrombosis

**DOI:** 10.1038/s41598-018-22780-1

**Published:** 2018-03-19

**Authors:** Teresa C. Assumpção, Daniella M. Mizurini, Dongying Ma, Robson Q. Monteiro, Sydney Ahlstedt, Morayma Reyes, Michail Kotsyfakis, Thomas N. Mather, John F. Andersen, Jan Lukszo, José M. C. Ribeiro, Ivo M. B. Francischetti

**Affiliations:** 1 Laboratory of Malaria and Vector Research, NIAID, National Institutes of Health, Bethesda, USA; 2 Institute of Medical Biochemistry, Federal University of Rio de Janeiro, Rio de Janeiro, Brazil; 3 Department of Pathology, Albert Einstein College of Medicine & Montefiore Medical Center, Bronx, NY USA; 4 Institute of Parasitology, Biology Center, Czech Academy of Sciences, České Budějovice, Czech Republic; 5 Rhode Island Center for Vector-Borne Disease, University of Rhode Island, Kingston, Rhode Island USA

## Abstract

Tick saliva is a rich source of modulators of vascular biology. We have characterized Ixonnexin, a member of the “Basic-tail” family of salivary proteins from the tick *Ixodes scapularis*. Ixonnexin is a 104 residues (11.8 KDa), non-enzymatic basic protein which contains 3 disulfide bonds and a C-terminal rich in lysine. It is homologous to SALP14, a tick salivary FXa anticoagulant. Ixonnexin was produced by ligation of synthesized fragments (51–104) and (1–50) followed by folding. Ixonnexin, like SALP14, interacts with FXa. Notably, Ixonnexin also modulates fibrinolysis *in vitro* by a unique salivary mechanism. Accordingly, it accelerates plasminogen activation by tissue-type plasminogen activator (*t*-PA) with Km 100 nM; however, it does not affect urokinase-mediated fibrinolysis. Additionally, lysine analogue ε-aminocaproic acid inhibits Ixonnexin-mediated plasmin generation implying that lysine-binding sites of Kringle domain(s) of plasminogen or t-PA are involved in this process. Moreover, surface plasmon resonance experiments shows that Ixonnexin binds *t*-PA, and plasminogen (K_D_ 10 nM), but not urokinase. These results imply that Ixonnexin promotes fibrinolysis by supporting the interaction of plasminogen with *t*-PA through formation of an enzymatically productive ternary complex. Finally, *in vivo* experiments demonstrates that Ixonnexin inhibits FeCl_3_-induced thrombosis in mice. Ixonnexin emerges as novel modulator of fibrinolysis which may also affect parasite-vector-host interactions.

## Introduction

The coagulation cascade is a series of limited proteolytic reactions, which culminates with thrombin generation and fibrin formation^1,2^. It is a tightly regulated process under control of 3 important anticoagulants including Tissue Factor Pathway Inhibitor (TFPI), Antithrombin (AT) and Activated Protein C (APC)^1,2^. Additionally, hemostasis is regulated by the fibrinolytic system that prevents excess thrombus formation by a mechanism where plasminogen activation by tissue-type plasminogen activator (*t-*PA) is amplified by cross-linked fibrin, which displays C-terminal lysine^3–5^. This interaction is mediated by lysine binding sites (LBS) present in the finger domain and Kringle 2 of *t-*PA, and one or more of the Kringle domains in plasminogen^6^. Also, C-terminal lysine generated by plasmin are particularly important as a positive feedback mechanism for the stimulation of fibrinolysis^6^. Fibrinolytic cascade and thrombus formation are regulated by at least 3 physiological inhibitors, including alpha2-antiplasmin (A2P), Plasminogen Activator Inhibitor (PAI)-1, and -2, and Thrombin Activatable Fibrinolysis Inhibitor (TAFI)^7,8^.

Salivary glands of blood-sucking arthropods display a notable repertoire of modulators of vascular biology. Several molecules have been characterized, including vasodilators, anticoagulants in addition to platelet aggregation and complement inhibitors^9–15^. These molecules block host response to injury therefore contributing to successful blood feeding. Surprisingly, few pro-fibrinolytic components have been identified in ticks, including metalloproteases^16,17^, an inhibitor of TAFI^18^ and plasminogen receptor enolase^19^. Fibrinolytic components are also important in parasite-vector-host interactions^20–26^. For instance, in Lyme Disease, a vector-borne disease transmitted by the tick *Ixodes scapularis*, fibrinolysis plays a critical role in *Borrelia sp*. dissemination, according to experiments with knock-out (KO) mice lacking plasminogen^27^.

More recently, next generation sequencing allowed a remarkable expansion in our understanding of the complexity of the salivary glands of blood-sucking arthropods^28,29^. However, some biological functions described in the salivary gland have not been associated with a specific protein. Likewise, several proteins coded by their corresponding salivary gland transcripts remain without a defined function. Among those is the “Basic-tail” family of proteins^30^ which is expanded in ticks and includes members such as SALP14, a Factor Xa (FXa) inhibitor^31^. We have identified Ixonnexin, a SALP14 homologue, as a novel fibrinolytic modulator by a mechanism involving binding to plasminogen and *t*-PA resulting in plasmin generation.

## Results

### Synthesis of Ixonnexin, a member of the “Basic-tail” family of salivary proteins

**Figure 1:**
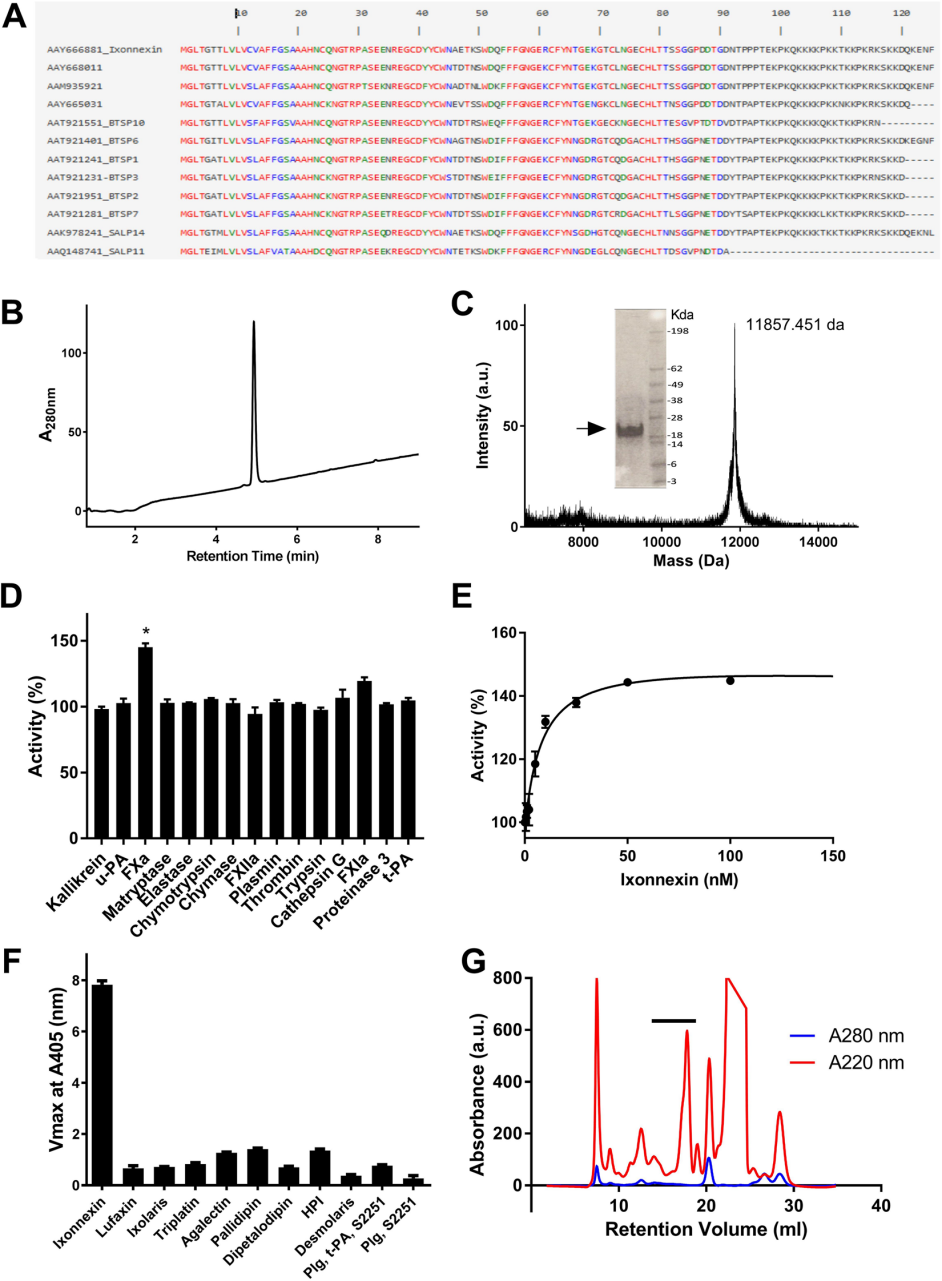
Synthesis and characterization of Ixonnexin, a novel modulator of fibrinolysis. (**A**) Clustal alignment of Ixonnexin (67083505; AAY66688.1) and other basic tail proteins from *Ixodidae*. (**B**) Ixonnexin was synthesized and the last purification step in RP-chromatography is shown. (**C**) Mass spectrometry of Ixonnexin. *Inset*, Purified Ixonnexin was loaded in a NuPAGE gel under reducing conditions. Gels were stained with Coomassie Blue. On the right, molecular mass markers are indicated. The arrow shows Ixonnexin. (**D**) Ixonnexin (250 nM) does not inhibit proteolytic activity of several enzymes involved in coagulation and inflammation^65^. (**E**) Ixonnexin increases catalytic activity of FXa (0.33 nM) measured with fluorogenic substrates. (**F**) Ixonnexin, but not other recombinant salivary proteins (1 μg/well), promotes plasmin generation. (**G**) Ixonnexin is expressed in tick saliva. The bar indicates the fraction where Ixonnexin were identified by proteomic analysis. A.u., arbitrary units. Representative experiments are shown.

Figure 1A shows the sequence alignment of several members of the “Basic-tail” family of salivary proteins from *Ixodes* sp. One of its members, named Ixonnexin, is 83% identical to SALP14, a FXa inhibitor^31^. These proteins have a molecular weight of ~11 kDa, and characteristically exhibit 6 cysteine residues and a C-terminal rich in lysine. Other members of this family have a naturally deleted C-terminal; for instance, TSLPI from *I. scapularis* is devoid of lysine-rich “basic tail”, and inhibits complement^32^. A phylogenetic tree of the Ixonnexin family members shows that it is particularly expanded in the salivary glands of *Ixodes sp* and other ticks (not shown).

In order to identify its function, Ixonnexin (gi 67083505) was initially expressed in *E. coli*. Given the very low yield, chemical synthesis was next attempted. The peptide was synthesized using native chemical ligation methodology with yield of approximately 32%, as described in the Material and Methods Section. After refolding, Ixonnexin was purified by reverse-phase chromatography, and one single peak was detected (Fig. 1B) with yield of 17%. Mass spectrometry analysis of Ixonnexin identified one single monomeric molecular mass of 11857.451 *Da*, which is in reasonable agreement with the theoretical mass of 11859.0 *Da* calculated for the correctly folded molecule with 3 oxidized disulphide bonds (Fig. 1C). SDS-PAGE shows that Ixonnexin migrates as a 20 kDa band, in denaturing and reducing conditions (Fig. 1C, inset). The molecular weight higher than expected is likely due to the basic p*I* 9.03 of the protein, which interferes with its migration. In order to verify whether Ixonnexin behaves as a monomer, or as higher-order oligomers, it was loaded onto a gel-filtration column previously calibrated with known molecular weight markers. The retention volume for Ixonnexin (11.91 ml) corresponds to a protein of 23.3 kDa which is consistent with a non-covalent dimer.

We tested Ixonnexin in screening assays in order to identify its biological activity. Ixonexin at high molar excess did not promote small fluorogenic substrates hydrolysis nor inhibited the activity of 15 enzymes involved in coagulation or inflammation (Fig. 1D). However, it enhances amidolytic activity of FXa in 45% with IC_50_ of 9.73 ± 2.5 nM (Fig. 1E) suggesting interaction with exosites^33,34^.

Because Ixonnexin contains several lysine residues in the C-terminal, and given the role of lysine in fibrin-mediated fibrinolysis^6,35^, it was hypothesized that Ixonnexin had a similar function, *i.e*., enhancing plasmin production by promoting the interaction of plasminogen with *t-*PA. Accordingly, Ixonnexin was immobilized in 96-well microplates and incubated with plasminogen and *t-*PA; plasmin production was measured by the rate of S-2251 hydrolysis. Figure 1F shows that Ixonnexin promotes plasmin production in a dose-dependent manner, indicating that it positively modulates fibrinolysis *in vitro*. In contrast, Ixonnexin did not promote plasmin formation when incubated with plasminogen and S-2251, in the absence of *t-*PA. These results imply that Ixonnexin is not a plasminogen activator. In order to verify if this effect was specific, several salivary proteins were immobilized and tested for plasmin generation in the presence of plasminogen and *t-*PA. Figure 1F shows that only Ixonnexin among several other recombinant proteins is biologically active.

In order to identify Ixonnexin family members in tick saliva, 100 μl of saliva was fractionated by gel-filtration chromatography (Fig. 1G). Tandem MS/MS revealed that several peptides compatible with Ixonnexin are present between the retention times of 15 and 18 min. Quantitative proteomic analysis determined that Ixonnexin is among the most abundant salivary proteins (not shown). This result is congruent with *I. scapularis* transcriptome, which also estimates Ixonnexin among the most abundant transcripts^28,29,36^.

### Ixonnexin is a potent modulator of fibrinolysis

**Figure 2:**
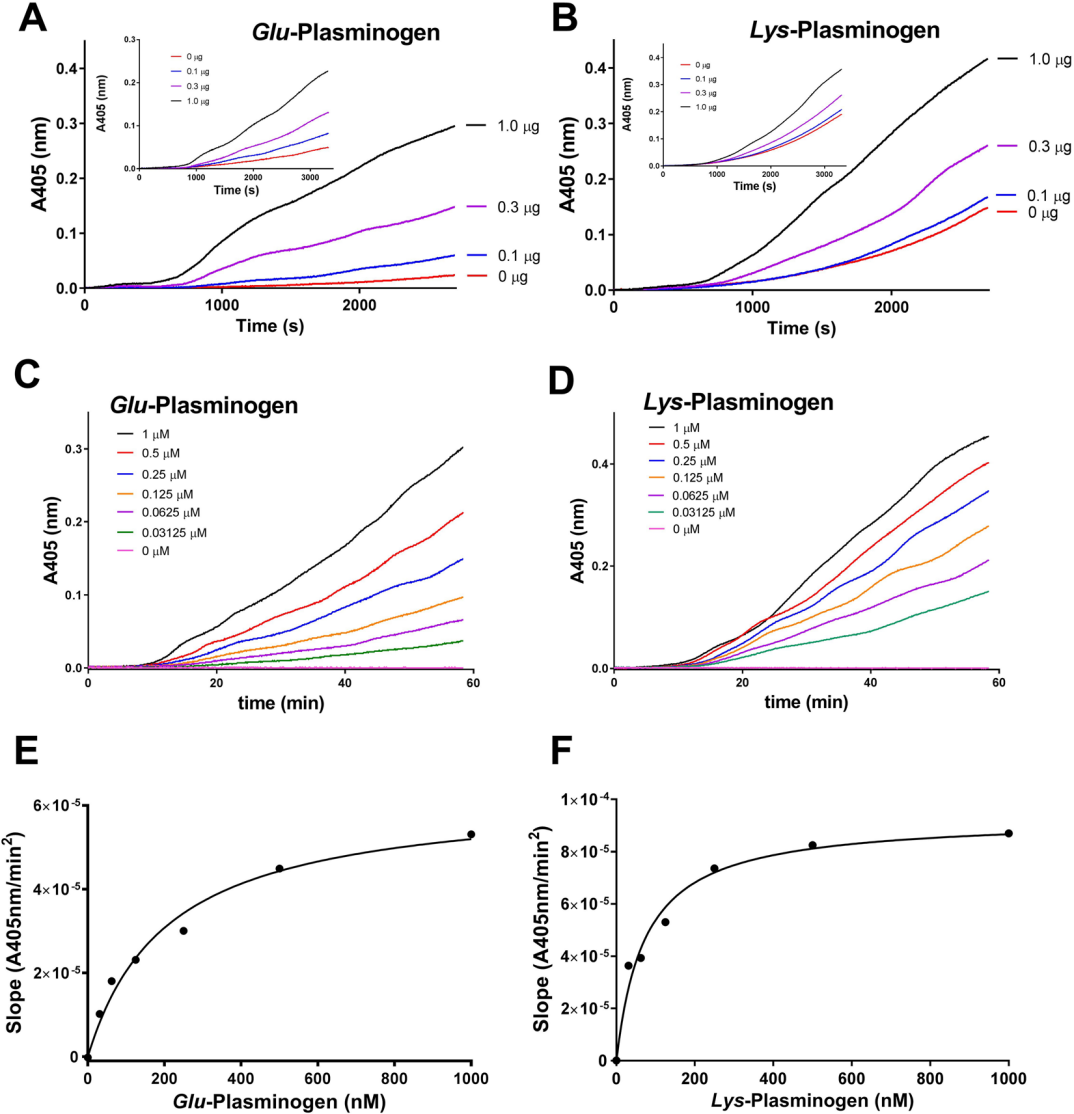
Ixonnexin promotes plasmin generation initiated with *t*-PA. (**A** and **B**) Ixonnexin (0–1 μg/ml) was immobilized in 96-well plates and incubated with *Glu*- or *Lys*-plasminogen (0.5 μM), respectively. Reactions were initiated with *t*-PA (2.5 nM for *Lys*-plasminogen, or 10 nM for *Glu*-plasminogen), and plasmin formation was detected with chromogenic substrate S2251 (250 μM). The inset shows plasmin generation in solution. (**C** and **D**) Ixonexin (0.5 μg/ml) mediated plasmin generation in the presence of increasing concentrations of *Glu*- and *Lys*-plasminogen, respectively. (**E** and **F**) Show the transformation of the data in (**C** and **D**) as the slope A405 nm/min^2^ and *Glu*- and *Lys*-plasminogen concentration, respectively. *Km* of the reaction using *Glu*- or *Lys*-plasminogen was calculated by non-linear regression, as indicated in Methods.

We determined the kinetics of Ixonnexin-mediated plasmin generation. Ixonnexin dose-dependently enhances *t-*PA mediated fibrinolysis in the presence of *Glu*-plasminogen (Fig. 2A) or *Lys*-plasminogen (Fig. 2B) with maximum effect attained at 1 μg/well (highest tested concentration). Ixonnexin in solution also promotes fibrinolysis, although at a slightly slower rate than observed with immobilized protein (Fig. 2A,B, insets). In order to determine the *Km* of the reaction, Ixonnexin was incubated with increasing concentrations of plasminogen and reactions started with *t-*PA. Figure 2C,D, respectively shows the progress curves for plasmin generation as a function of time in the presence of *Glu*- or *Lys*-plasminogen, respectively. Figure 2E,F shows the transformation of the data as a function of the slope (A405 nm/min^2^) for *Glu*- and *Lys*-plasminogen, respectively. The calculated *Km* are 207.6 ± 40.03 nM and *Vmax* 2.6 μM/min^2^ for *Glu*-plasminogen, and 72.83 ± 13.87 nM and *Vmax* of 3.1 μM/min^2^ for *Lys*-plasminogen, respectively. This is 1000 lower than the *Km* calculated for *t*-PA induced plasminogen activation in the absence of fibrin, which is in the range of 19–65 μM^4^.

### ε–Aminocaproic acid (ε-ACA) interferes with Ixonnexin activity

**Figure 3:**
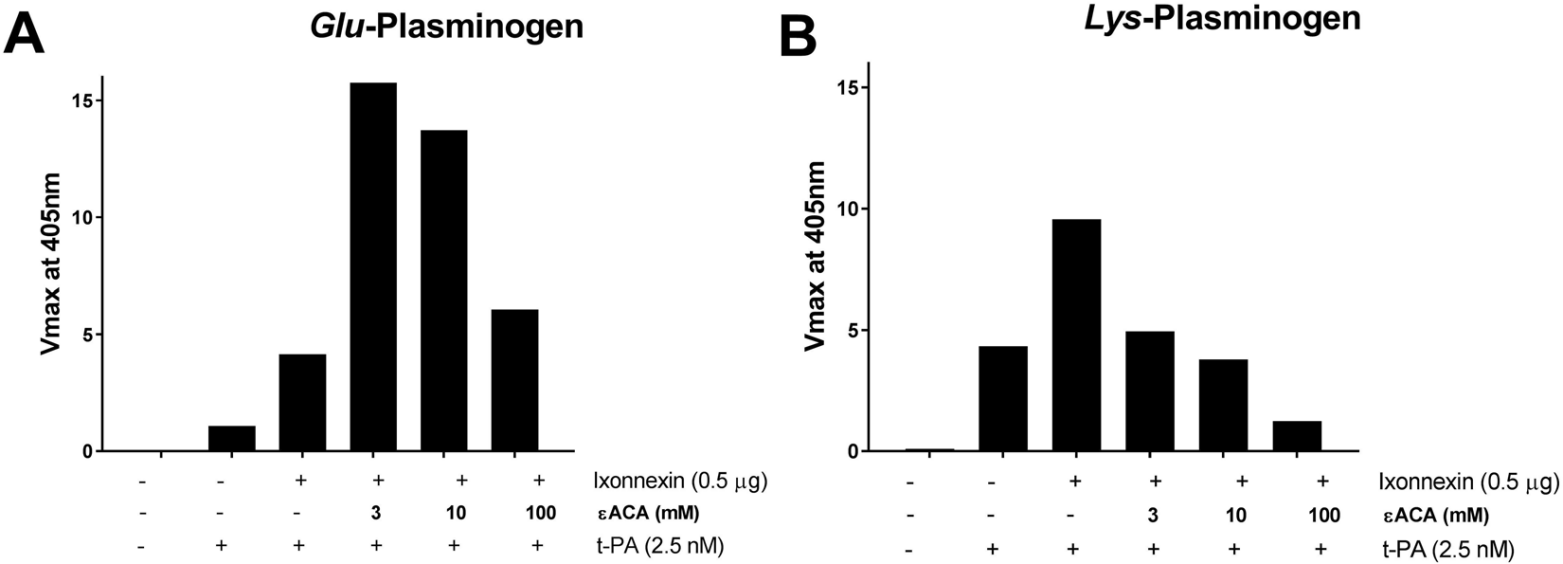
Effects of ε-aminocaproic acid on Ixonnexin-mediated plasmin formation. Immobilized Ixonnexin (0.5 μg/ml) was incubated with (**A**) *Glu*- or (**B**) *Lys*-plasminogen in the presence of ε-ACA. Reactions were initiated with *t*-PA (2.5 nM for *Lys*-plasminogen, or 10 nM for *Glu*-plasminogen), and plasmin formation was detected with chromogenic substrate S2251 (250 μM). Experiment was done in triplicate and SE bar is invisible.

ε-ACA is a lysine analogue with antifibrinolytic properties that prevents the interaction of plasminogen Kringle domain with fibrin and inhibits *t-*PA activity^6,37^. Figure 3A shows that ε-ACA increases *Glu*- plasminogen activation by *t-*PA in the presence of Ixonnexin at 3 and 10 mM, and an inhibitory effect is observed at 100 mM. This increase in activity has been explained by ε-ACA opening the conformation of *Glu*-plasminogen upon binding to LBS and enhancing activation by plasminogen activators such as urokinase^6,37^ and staphylokinase^38^. In contrast, when *Lys*-plasminogen is added as substrate, ε-ACA completely blocks plasmin generation at 3 mM (Fig. 3B). These results suggest that LBS plays a role in the interaction of Ixonnexin with components of the fibrinolytic system.

### Ixonnexin displays high-affinity binding to plasminogen and *t*-PA

**Figure 4:**
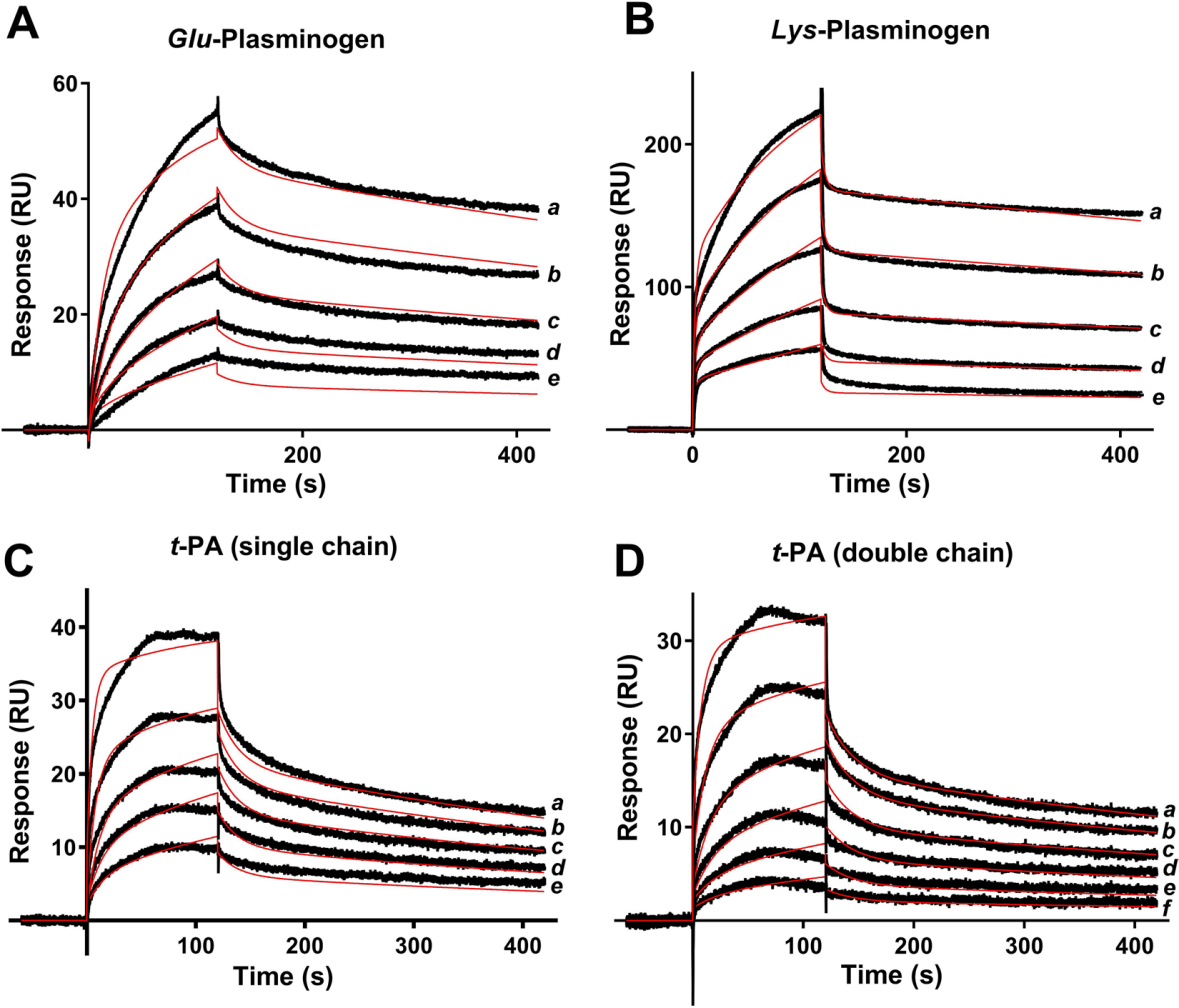
Ixonnexin binds to plasminogen and *t-*PA. Ixonnexin was immobilized in a sensor chip and the following analytes were tested: (**A**) *Glu*-plasminogen (15–250 nM); (**B**) *Lys*-plasminogen (31–500 nM); (**C**) Single chain *t-*PA (7.8–125 nM); (**D**) Double chain *t*-PA (7.8–250 nM). Dissociation of the complexes was monitored for 600 seconds, and a two-state reaction binding model was used to calculate kinetic parameters. Representative sensograms are shown in black, and fitting of the data points is depicted in red. Representative experiments are shown.

Kinetics of Ixonnexin interaction with plasminogen and *t-*PA were studied by SPR. Ixonnexin was immobilized in a carboxymethylated dextran sensor chip, and components of the fibrinolytic system used as analytes. Typical sensograms are shown in Fig. 4A–D. Best global fit was attained using 2-state model (conformational change) for plasminogen and *t*-PA with *K*_*D*_ in the low nanomolar range. Although the fitting of the SPR experiments indicated a single binding site between ixonnexin and *t*-PA, we cannot exclude a second binding site with a lower affinity, suggested by the curve fitting at the highest plasminogen concentrations (Fig. 4). Nonetheless, the experiment indicates a high affinity interaction between Ixonnexin and *t*-PA. In addition, other models (*e.g*. 1:1 interaction, solution heterogeneity, surface heterogeneity, bivalent analyte) did not yield better fitting or lower χ^2^. Table 1 summarizes the kinetic values, Rmax and χ^2^ calculated for each interaction.

**Table 1:** Kinetics of Ixonnexin interaction with components of the fibrinolytic system.

	*ka1* (M^−1^s^−1^)	*kd1* (s^−1^)	*ka2* (s^−1^)	*kd2* (s^−1^)	*K*_***D***_ (nM)	R_max_	χ^2^
*Glu*-plasminogen	1.23 × 10^5^	5.7 × 10^−2^	2.2 × 10^−2^	9.9 × 10^–4^	19.52	61.8	3.5
*Lys*-plasminogen	3.39 × 10^5^	2.3 × 10^–1^	2.4 × 10^−2^	5.1 × 10^−4^	14.45	248.0	15.1
Single-chain *t-*PA	4.13 × 10^5^	3.1 × 10^−2^	9.7 × 10^−3^	1.5 × 10^−3^	4.67	36.8	1.3
Two-chain *t-*PA	1.20 × 10^6^	4.3 × 10^−1^	1.1 × 10^−2^	1.8 × 10^−3^	10.26	31.4	0.7
Plasmin	1.48 × 10^6^	2.9 × 10^−1^	—	—	197.40	305.8	27.7

### Ixonnexin does not bind urokinase

**Figure 5:**
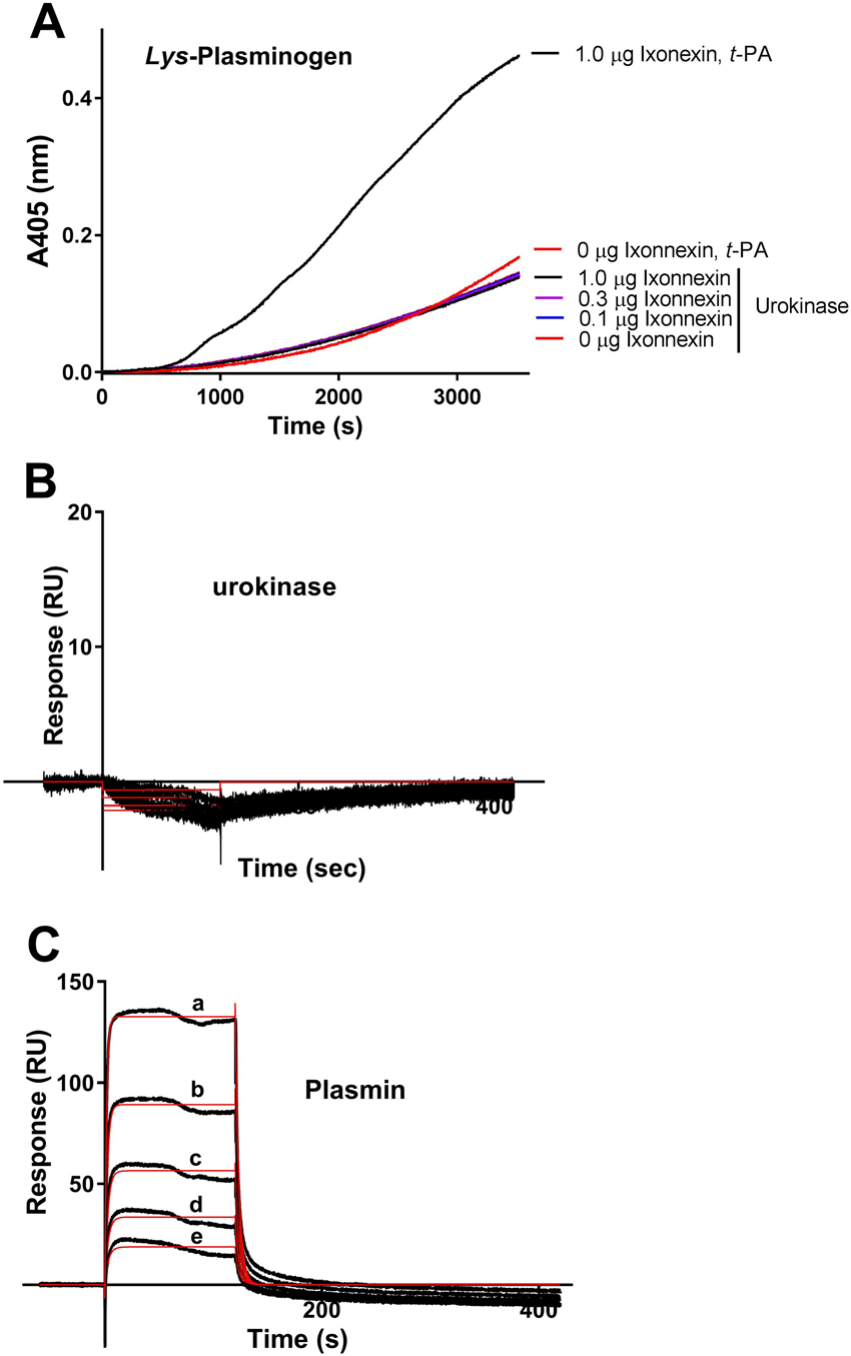
Ixonnexin does not bind to urokinase. (**A**) Ixonnexin was immobilized in 96-well plates as indicated and incubated with *Lys*-plasminogen. Reactions were initiated with urokinase (0.1 nM) or *t*-PA (2.5 nM), and plasmin formation was detected with chromogenic substrate S2251 (250 μM). SPR experiments: Ixonnexin was immobilized in a sensor chip and (**B**) urokinase (15–250 nM) or (**C**) plasmin (15–250 nM) were tested as analytes. Dissociation of the complexes was monitored for 600 seconds, and a two-state reaction binding model was employed for urokinase and Langmuir equation (1:1) for plasmin. Representative sensorgrams are shown in black, and fitting of the data points is depicted in red. Representative experiments are shown.

Urokinase has one single Kringle domain, and differs from plasminogen and *t-*PA, which have 5 and 2 Kringle domains, respectively. Urokinase also poorly interacts with fibrin, and does not rely on a colocalization mechanism like *t*-PA and fibrin^6,35^. Figure 5A shows that urokinase generates plasmin in the presence *Lys*-plasminogen, comparable to *t*-PA initiated reactions. However, immobilized Ixonnexin did not accelerate fibrinolysis, in contrast to reactions started with *t-*PA. Corroborating with these findings, immobilized Ixonnexin does not bind urokinase (Fig. 5B). Figure 5C also shows that Ixonnexin did not display high affinity binding to plasmin (Table 1), the product of the reaction.

### Ixonnexin exhibits antithrombotic activity

**Figure 6:**
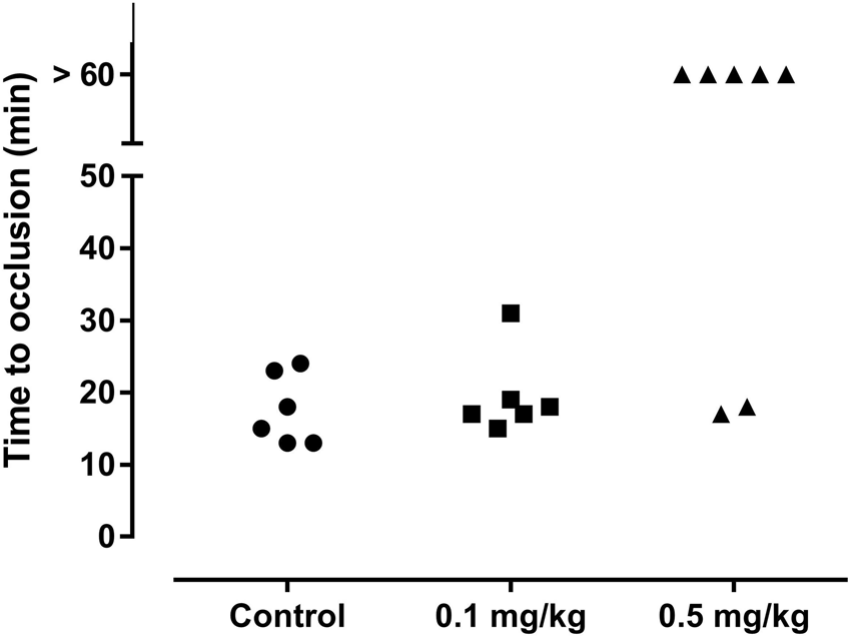
Ixonnexin exhibits antithrombotic activity *in vivo*. (**A**) A paper filter imbibed with 7.5% FeCl_3_ was applied to carotid artery, and blood flow was monitored with a perivascular flow probe for 60 minutes or until stable occlusion took place. Fifteen minutes before injury, Ixonnexin was injected into the caudal vein of the mice. Each symbol represents 1 animal (*n* = 7). *p* < 0.001 (0.5 mg/Kg *vs* control).

To test whether Ixonnexin displays antithrombotic activity, we employed a mouse model of thrombosis in which FeCl_3_ induces carotid artery injury. Thrombus formation was estimated using a Doppler flow probe that allows monitoring of carotid blood flow for 60 minutes or until complete occlusion takes place. Figure 6 shows that the time to occlusion was not significantly different between control and mice treated with 100 µg/kg Ixonnexin; however, mice treated with 500 µg/kg were resistant to arterial occlusion. In these cases, occlusion did not take place before 60 minutes for most animals.

## Discussion

Ixonnexin, a member of the “Basic-tail” family of salivary proteins, is among the most abundant proteins in *I. scapularis* salivary glands. In this study, Ixonnexin was entirely obtained by chemical synthesis using native chemical ligation methodology followed by refolding and purification, resulting in a protein with a single molecular mass. Ixonnexin is 83% identical to SALP14, a tick FXa anticoagulant expressed as a fusion protein with maltose-binding protein (MBP)^31^. In our experimental conditions, Ixonnexin, like MBP-SALP14, interacts with FXa. In contrast to MBP-SALP14, however, it augments the amidolytic activity of FXa, suggesting allosteric interactions mediated by exosites^33,34,39^. These results provide additional evidence to conclude that Ixonnexin and SALP14 are anticoagulants. Notably, Ixonnexin was found to enhance fibrinolysis *in vitro* by a unique salivary mechanism. Accordingly, Ixonnexin supports the interaction of plasminogen and *t-*PA through formation of an enzymatically productive ternary complex. This assumption is based on (*i*) presence of several internal lysine residues in the C-terminus of Ixonnexin, (*ii*) high-affinity binding of Ixonnexin to plasminogen and *t*-PA, (*iii*) oligomerization as a non-covalent dimer, which may facilitate interaction with reactants, (*iv*) *in vitro* functional assays with purified proteins showing enhanced plasmin generation, (*v*) inhibition of this activity by ε-ACA, a lysine analogue, and (*vi*) Ixonnexin’s antithrombotic activity *in vivo*. Therefore, Ixonnexin is not a plasminogen activator, and differs from tick metalloproteases which promotes clot degradation by an enzymatic mechanism^16^, from “Tick Carboxypeptidase Inhibitor” (TCI) that targets TAFI through enzyme-inhibitor [EI] complex formation^18^, and from enolase which operates as a surface-bound plasminogen receptor^19,40^.

Despite obvious differences in the primary sequence, it is worth noting that Ixonnexin mimics polymerized fibrin in several respects. Accordingly, fibrin displays C-terminal lysine residues that function as a high-affinity binding site for the LBS in the Kringle domains of plasminogen and *t-*PA^6,41^. As a result, fibrin-mediated activation of fibrinolysis promotes a decrease in the *Km* from 19–65 μM to 0.14 μM for plasminogen^4^. Similarly, Ixonnexin decrease the *Km* of the reaction in ~1000 fold^4^. Moreover, ixonnexin, like fibrin^6,41^, does not enhance urokinase-mediated fibrinolysis. We propose that Ixonnexin operates as a “soluble fibrin” promoting assembly of fibrinolysis, as reported for annexin-2/S100 complex^42,43^ and Prion Protein^44^ among others proteins^19,40^.

Ixonnexin binds plasminogen which is present in plasma at high concentrations (1.5–2 μM) approximately 100 times higher than the *K*_*D*_ (~10 nM) of the interaction. This finding is relevant for Ixonnexin’s mechanism of action suggesting that it remains bound to plasminogen, which has plasma half-life of 48 hours^35^. The zymogen-binding property of Ixonnexin resembles Ixolaris mechanism of action, which binds to FX exosite^39^ and serves as a scaffold for inhibition of FVIIa/TF complex, with a long lasting antithrombotic effect of 24 hrs^45,46^. Ixonnexin therefore is another example of a protein with more than one target and distinct mechanisms of action. In fact, multifunctional proteins are increasingly recognized in salivary gland secretions^47–51^.

*In vivo* experiments show that Ixonnexin given intravenously to mice 15 minutes before carotid artery injury with FeCl_3_ prolongs time to occlusion. This indicates that Ixonnexin interferes with thrombus formation. Nevertheless, additional studies are required to understand the relative contribution of anticoagulant *vs* pro-fibrinolytic activity determined here *in vitro*, and the relevance of these findings for Ixonnexin’s antithrombotic activity *in vivo* and whether it is associated with bleeding.

Our results are pertinent in tick biology since a potent fibrinolytic enzyme is present in tick saliva^16^. Because a plasminogen activator has not been identified in the saliva incubated with plasminogen and chromogenic substrate S-2251 (not shown), it is conceivable that host *t*-PA employs Ixonnexin, not fibrin, as a cofactor to initiate fibrinolysis. Also, Ixonnexin C-terminal residue is phenylalanine while TAFI, a carboxypeptidase, has specificity for lysine^7,8,52^. Additionally, Ixonnexin is abundantly expressed in *Ixodes sp*, according to transcriptome^28–30,36^, gene expression^36,53^ and proteome studies (Fig. 1G), suggesting that it may reach high concentrations necessary to inhibit coagulation and/or stimulate fibrinolysis in the feeding cavity. Of note, D-dimers are found in *I. scapularis* ticks feeding on the host, indicating that fibrinolysis mediated by plasmin does occur at sites of attachment^54^. Moreover, silencing of SALP14 in the salivary gland, which presumably also has pro-fibrinolytic activity given its conserved “basic tail”, is accompanied by reduced ability of ticks to feed resulting in a decline in engorgement weights^55^. Finally, Ixonnexin family is expanded in *Amblyomma sp*^56^*, Hyalomma sp*^57^*, Rhipicephalus sp*^58^*, Antricola sp*^59^ *and Ornithodorus sp* ticks^28^. Altogether, these results highlight the importance of the “basic-tail” family of salivary proteins in tick biology. Furthermore, the fibrinolytic system is critical for *Borrelia sp* infection since mice lacking plasminogen have impaired dissemination^27^ raising the possibility that Ixonnexin contributes to parasite-vector-host interactions^20–26^. We speculate that mosquitoes, bugs and sandflies may rely in unknown function salivary proteins in order to promote fibrinolysis by a similar mechanism described here for Ixonnexin; this activity may contribute to parasite transmission to the host and/or the vector.

Ixonnexin adds to the repertoire of modulators of hemostasis present in *I. scapularis* saliva^9–15^. Identification of Ixonnexin as a novel fibrinolysis modulator is relevant to study the participation of plasmin in ischemic events, tumor growth, metastasis^5,60,61^ and *Borrelia* sp. transmission^22–26^. Ixonnexin may be useful as a prototype for the development of novel drugs with therapeutic potential.

## Material and Methods

### Ethical Statement

All animal care and experimental protocols were conducted following the NIH Guide for the Care and Use of Laboratory Animals (ISBN 0-309-05377-3) guidelines and the Committee for Evaluation of Animal Use for Research from the Federal University of Rio de Janeiro, CAUAP-UFRJ under registry #IBQM/081-05/16. Technicians dedicated to the animal facility carried out all aspects related to mouse husbandry under strict guidelines for careful and consistent care and handling of the animals.

### Reagents

*Glu*- and *Lys*-plasminogen, plasmin, low molecular weight urokinase and single or double chain *t*-PA were from Enzyme Research Laboratories (South Bend, IN) or Innovative Research (Novi, MI). Activated partial thromboplastin time (aPTT; STA-PTT Automate) and prothrombin time (PT; Neoplastine CI Plus) reagents were from Diagnostica Stago (Asnières, France). S-2251 (H-D-Valyl-L-leucyl-L-lysinep-Nitroaniline dihydrochloride) was obtained from Diapharma (West Chester, OH). ε-Aminocaproic acid (ε-ACA), 4-mercaptophenylacetic acid, trifluoroacetic acid (TFA), triisopropylsilane (TIS), diisopropylcarbodiimide (DIC), 3,6-Dioxa-1,8-octane-dithiol (DODT), 4-methylpiperidine, methyl t-butyl ether (MTBE) and tris(2-carboxyethyl)phosphine hydrochloride (TCEP) were from Sigma (Saint Louis, MO). HBTU, N,N-diisopropylethylamine (DIPEA) and 1,1,1,3,3,3-hexafluoroisopropanol (HFIP) were from Chem-Impex International (Wood Dale, IL). Fmoc-amino acid for peptide synthesis were from Midwest Biotech Inc (Fishers, IN). Dimethylformamide (DMF) and N-methylpyrrolidone (NMP) were from AGTC Bioproducts (Framingham, MA).

### Tick saliva and Mass spectrometry

*I. scapularis* ticks were reared at the University of Rhode Island and were fed on rabbits. Saliva collection and salivary gland homogenates were obtained as reported^62^. Proteomic analysis of saliva was performed as described^63^.

### Synthesis of Ixonnexin

Full length Ixonnexin (gi 67083505) has 21 residues in the signal peptide with cleavage site between positions Ala^21^ and His^22^: AAA-HN (Signal P 4.1 server). Mature Ixonnexin (104 residues) was synthesized by ligation of separately synthesized fragment (51–104) with the C-terminal thioester of fragment (1–50), utilizing Native Chemical Ligation (NCL)^64^ methodology followed by folding of the ligation product.

### Ixonnexin fragment (51–104)

It was synthesized using an automated peptide synthesizer, model 433 A (Applied Biosystems, Fullerton, CA, USA) with Fmoc strategy and HBTU/DIPEA as the coupling reagent. Novabiochem Fmoc-Phe-Wang-LL resin (0.20 mmol) (EMD Millipore, division of Merck KGaA, Darmstadt, Germany) was used as the solid phase. The side-chain protecting groups used in synthesis were Trt for Asn, Cys, Gln, and His; OtBu for Glu and Asp; Pbf for Arg; and tBu for Ser, Thr, and Tyr. The coupling reaction time was 1 h, and 4-methylpiperidine (20%)/N-methylpyrrolidone was used to remove the Fmoc group at every step. Peptide resin was washed with N-methylpyrrolidone and dichloromethane and dried *in vacuo* to yield the protected peptide-resin. The peptide resin was treated with a cleavage mixture of trifluoroacetic acid/water/Triisopropylsilane/3,6-Dioxa-1,8-octane-dithiol (92.5∶2.5∶2.5∶2.5, v/v/v/v; 40 ml) for 2.5 h to remove protecting groups and peptide from the resin. After filtration of the exhausted resin, the solvent was concentrated *in vacuo* and the residue was triturated with methyl t-butyl ether. The solid peptide was filtered off, washed with methyl t-butyl ether, and vacuum dried. The crude peptide was purified by preparative reversed-phase high-performance liquid chromatography (HPLC), and purity grade was checked by analytical HPLC analyses and mass spectrometry using a matrix-assisted laser desorption ionization time-of-flight mass spectrometer Axima CFR + (Shimadzu Scientific Instruments. Columbia, MD, USA). Pure fractions were combined, frozen, and lyophilized to afford Ixonnexin (51–104) peptide. Peptide was 95% pure (MALDI-TOF MS: m/z calculated 6035.9, found 6037.1 [M + H^+^]).

### Ixonnexin fragment (1–50) thioester

It was synthesized using an automated peptide synthesizer, model 433 A (Applied Biosystems, Fullerton, CA, USA) with Fmoc strategy and HBTU/DIPEA as the coupling reagent. Novabiochem Fmoc-Thr(tBu)-2-ClTrt resin (0.30 mmol) (EMD Millipore, division of Merck KGaA, Darmstadt, Germany) was used as the solid phase. The side-chain protecting groups used in synthesis were Trt for Asn, Cys, and His; OtBu for Glu and Asp; Pbf for Arg; and tBu for Ser, Thr, and Tyr. The coupling reaction time was 1 h, and 4-methylpiperidine (20%)/N-methylpyrrolidone was used to remove the Fmoc group at every step. Following the final Fmoc deprotection, the N-terminal amino group was protected with Boc group by reacting the peptide resin with di-t-butyl dicarbonate and DIPEA in dimethylformamide (DMF). After washing with DMF and t-butyl methyl ether and drying in vacuum, the fully protected peptide resin was treated with the HFIP/dichloromethane (1:3, v/v, 3 × 15 min) mixture, collecting all filtrates. The combined filtrates were concentrated in vacuum and the residue was triturated with methyl t-butyl ether (30 mL) to yield a white precipitate. Filtration, washing with methyl t-butyl ether and drying afforded a fully protected peptide fragment with a free C-terminal carboxy function. This solid peptide was treated with ethyl ß-mercaptoacetate (TCI America, Portland, OR), diisopropylcarbodiimide (DIC), and DIPEA (5 equiv.each) in DMF for 18 hrs followed by removal of the solvent in high vacuum and trituration of the oily residue with methyl t-butyl ether and separation of the precipitate by filtration. After drying in vacuum, the crude thioester was treated with a cleavage mixture of trifluoroacetic acid(TFA)/water/triisopropylsilane/3,6-Dioxa-1,8-octane-dithiol (DOTH) (92.5∶2.5∶2.5∶2.5, v/v/v/v; 30 ml) for 2.0 h to remove protecting groups. After concentration and trituration with the cold ethyl ether, the crude solid peptide thioester was filtered off, washed with ethyl ether and dried. The pure thioester of Ixonnexin (1–50) fragment was isolated by preparative reverse-phase HPLC and its purity checked by HPLC analyses and mass spectrometry using a matrix-assisted laser desorption ionization time-of-flight mass spectrometer Axima CFR + (Shimadzu Scientific Instruments). Pure fractions were combined, frozen, and lyophilized to afford pure C-terminal thioester (MALDI-TOF MS: m/z calculated 5965.4, found 5966.8 [M + H^+^]).

### Ligation of Ixonnexin fragment (1–50) thioester and Ixonnexin fragment (51–104)

Purified Ixonnexin (1–50) thioester 2.0 µM and Ixonnexin (51–104) 2.3 µM were dissolved in 1.9 mL of 6 M guanidine hydrochloride/200 mM PBS buffer containing 4-mercaptophenylacetic acid (Sigma-Aldrich, St.Louis, 68 mg) and tris(2-carboxyethyl)phosphine hydrochloride (TCEP, Sigma-Aldrich, St.Louis, 68 mg). The pH of the mixture was adjusted to 7.1 with 6 M sodium hydroxide and the resultant solution was kept at room temperature, monitoring ligation progress by analytical HPLC using a gradient of acetonitrile/water with UV monitoring at 215 nm. After 24 hrs the reaction mixture was acidified with diluted (~2%) TFA and the product was isolated by preparative reverse-phase HPLC and its purity checked by HPLC analyses and MALDI-TOF mass spectrometry. Pure fractions were combined, frozen, and lyophilized to afford pure unfolded Ixonnexin 1–104 with 32% theoretical yield (MALDI-TOF MS: m/z calculated 11867.1, found 11868.0 [M + H^+^]).

### Folding of Ixonnexin

Linear, purified peptide Ixonnexin (1–104, mature protein) was dissolved in 6 M guanidin•HCl/200 mM PBS at a concentration of 2 mmol, and the solution was introduced by a Harvard Apparatus “Elite 11” through a syringe, to a 30 times larger volume of the stirred solution of degassed, 50 mM Tris/1 mM EDTA, pH∼8, containing reduced glutathione and oxidized glutathione at concentrations of 1.6 mM and 0.2 mM, respectively. The progress of folding was monitored by HPLC using a gradient of acetonitrile/water with UV monitoring at 215 nm. After 3 h, the reaction mixture was acidified with 2% trifluoroacetic acid to pH 5. The folded peptide was isolated by preparative reverse-phase HPLC and its purity checked by HPLC analyses and mass spectrometry using a matrix-assisted laser desorption ionization time-of-flight mass spectrometer Axima CFR + (Shimadzu Scientific Instruments). Pure fractions were combined, frozen, and lyophilized to afford pure, folded peptide with 17% theoretical yield. Extinction coefficient of Ixonnexin at 280 nm is 15845 (all disulfide bonds); A280 nm/cm (1 mg/mL), 1.33. (MALDI-TOF MS: m/z calculated 11861.0, found 11863.0 [M + H^+^]).

### Gel-filtration chromatography

Ixonnexin was loaded onto a Superdex 75 10/300 (GE Healthcare) column equilibrated in 20 mM Tris-HCl, NaCL 0.15 M, pH 8 with a flow of 0.5 mL/min and connected to AKTA purifier system (Amersham Biosciences, Uppsala, Sweden) Pharmacia HPLC system. Protein was detected by peak absorbance at 280 and 220 nm. Column was calibrated with the following recombinant salivary proteins with respective mol wt and retention volumes: D7 (26.7 kDa, 11.64 ml), 9G11 (14.4 kDa, 12.90 ml), FS50 (8.1 kDa, 13.49 ml). The log of mol wt markers was plotted *vs* the retention volumes resulting in a straight line where retention volumes are interpolated.

### PAGE

The samples were treated with 4 × NuPAGE lithium dodecyl sulfate sample buffer and analyzed in NuPAGE 4% to 12% gels with 2-(N-morpholino)ethanesulfonic acid running buffer.

### Plasminogen activation assays

Ixonnexin, or other recombinant salivary proteins (in 100 μl PBS, 0–1 μg/well) were immobilized overnight in 96-well plates (Costar). Wells were washed 3 times in TBS containing 0.03% BSA and 5 mM CaCl_2_ (TBS-BSA buffer). *Glu*- or *Lys*-plasminogen (0.5 μM) and S2251 (250 μM) was added to the wells and incubated for 30 min at room temperature. Then, single chain *t-*PA (10 nM for *Glu*-plasminogen, or 2.5 nM for *Lys*-plasminogen) or urokinase (0.1 nM for *Lys*-plasminogen) were added to start reactions, in a final volume of 100 μl. Plasmin generation was detected at A405 nm at 37 °C, using ELISA reader as described^65^. Initial rates of plasmin generation were calculated using linear regression analysis of plots of absorbance (A405 nm) *vs* time (min)^2^, as described^66–68^ using GraphPad Prism (La Jolla, CA). A standard curve for substrate hydrolysis was performed with *p*-nitroanilide (Sigma). In some experiments, ε-ACA was incubated with the reactants.

### Surface Plasmon Resonance

All surface plasmon resonance (SPR) experiments were carried out in a T100 instrument (Biacore Inc, Uppsala, Sweden) following the manufacturer’s instructions. For immobilization using an amine coupling kit (Biacore), carboxymethylated dextran chips were activated with 1-ethyl-3-(dimethylaminopropyl) carbodiimide, and N-hydroxysuccinimide before injection of Ixonnexin (10 µg/mL) in acetate buffer, pH 5.5. Remaining activated groups were blocked with 1 mol/L ethanolamine, pH 8.5, resulting in a final immobilization of 726.2 RU. Kinetic experiments were carried out by injecting plasminongen, urokinase or *t*-PA for a contact time of 120 seconds at a flow rate of 30 µL/minute at 25 °C as described^65^. After subtraction of the contribution of the bulk refractive index and non-specific interactions with the CM5 chip surface, the individual association (ka) and dissociation(kd) rate constants were obtained by global fitting of the data using the two-state reaction (conformational change) interaction model in BIAevaluation software (Biacore Inc.). In this model, the analyte (A) binds to the ligand (B) to form an initial complex (AB) and then undergoes subsequent binding or conformational change to form a more stable complex (AB*). Model parameters are: *ka*1, association rate constant for analyte binding; *kd*1, dissociation rate constant for analyte from the complex; *ka*2, forward rate constant for the conformational change; *kd*2, reverse rate constant for the conformational change. These values were then used to calculate the dissociation constant (K_D_). The values of mean squared residual obtained were not significantly improved by fitting data to models that assumed other interactions. Conditions were chosen so that the contribution of mass transport to the observed values of K_D_ was negligible. In addition, the models in the T100 evaluation software fit for mass transfer coefficient to mathematically extrapolate the true *ka* and *kd*.

### FeCl_3_-Induced Artery Thrombosis

Thrombus formation was induced by applying a piece of filter paper (1 × 2 mm) saturated with 7.5% FeCl_3_ solution on the adventitial surface of the artery for 3 minutes. After exposure, the filter paper was removed, and the vessel was washed with sterile normal saline. Carotid blood flow was continuously monitored for 60 minutes or until complete occlusion (0 flow for at least 10 seconds) occurred as reported^69^.

### Protease Inhibition Assays

This was performed essentially as described^65,70^.

### Statistical analysis

Results are expressed as means ± SE. Statistical differences among the groups were analyzed by *t* test. Significance was set at P ≤ 0.05 (Graph-Pad Prisma software, La Jolla, CA).
